# Neuropharmacological Effects of Terpenoids on Preclinical Animal Models of Psychiatric Disorders: A Review

**DOI:** 10.3390/antiox11091834

**Published:** 2022-09-18

**Authors:** Tamanna Jahan Mony, Fazle Elahi, Ji Woong Choi, Se Jin Park

**Affiliations:** 1 Agriculture and Life Science Research Institute, Kangwon National University, Chuncheon 24341, Korea; 2 Department of Food Science and Biotechnology, Kangwon National University, Chuncheon 24341, Korea; 3 College of Pharmacy, Gachon University, Incheon 21936, Korea; 4 School of Natural Resources and Environmental Sciences and Agriculture and Life Science Research Institute, Kangwon National University, Chuncheon 24341, Korea

**Keywords:** neuropharmacological effect, terpenoid, preclinical, animal model, psychiatric disorders

## Abstract

Terpenoids are widely distributed in nature, especially in the plant kingdom, and exhibit diverse pharmacological activities. In recent years, screening has revealed a wide variety of new terpenoids that are active against different psychiatric disorders. This review synthesized the current published preclinical studies of terpenoid use in psychiatric disorders. This review was extensively investigated to provide empirical evidence regarding the neuropharmacological effects of the vast group of terpenoids in translational models of psychiatric disorders, their relevant mechanisms of action, and treatment regimens with evidence of the safety and psychotropic efficacy. Therefore, we utilized nine (9) electronic databases and performed manual searches of each. The relevant data were retrieved from the articles published until present. We used the search terms “terpenoids” or “terpenes” and “psychiatric disorders” (“psychiatric disorders” OR “psychiatric diseases” OR “neuropsychiatric disorders” OR “psychosis” OR “psychiatric symptoms”). The efficacy of terpenoids or biosynthetic compounds in the terpenoid group was demonstrated in preclinical animal studies. Ginsenosides, bacosides, oleanolic acid, asiatic acid, boswellic acid, mono- and diterpenes, and different forms of saponins and triterpenoids were found to be important bioactive compounds in several preclinical studies of psychosis. Taken together, the findings of the present review indicate that natural terpenoids and their derivatives could achieve remarkable success as an alternative therapeutic option for alleviating the core or associated behavioral features of psychiatric disorders.

## 1. Introduction

Terpenoids are widely distributed in plants, microorganisms, fungi, marine organisms, animals, sedimentary rocks, and oils [1]. These compounds are structurally diverse, biosynthetically formed natural products and are sometimes referred to as “terpenes.” The term “terpenoid” denotes a compound that contains an integral number of C5 units and is derived from the basic branch C5 unit isoprene (2-methyl-1,3-butadiene) [2]. The diverse structural variations among terpenoids give this large group of molecules a wide range of potent biological activities in areas such as cell membrane construction, signal transduction, immunomodulation, inflammation control, antioxidation, and inhibition of several enzymes [1,3,4].

Terpenoids and their semisynthetic derivatives may represent promising neuroprotective agents against several neurological and cognitive dysfunctions. Celastrol, ginsenosides, oleanolic acid, ursolic acid, asiatic acid, erythrodiol, and some triterpenoid saponins have been studied for years and have shown efficacy in protecting the brain against processes including neuroinflammation and oxidative stress [5,6]. Many more have also attracted interest in recent years, including lupeol, rosmarinic acid, resveratrol, betulinic acid, pomolic acid, maslinic acid, uvaol, tormentic acid, and erythrodiol [5]. These compounds are found in higher plants, including common edible and nonedible plants, and may occur as free compounds, conjugates, or saponins (with one or more sugar units) [5,7,8]. These terpenoids have been used for centuries in traditional medicine to improve memory and cognitive function. Some of them are in preclinical or clinical trials, and some have already been approved for use in humans. The diverse structures and functions of terpenoids have sparked interest in investigating the commercial use of these compounds, emphasizing their practical importance as alternative medicines for psychiatric disorders.

**Figure 1:**
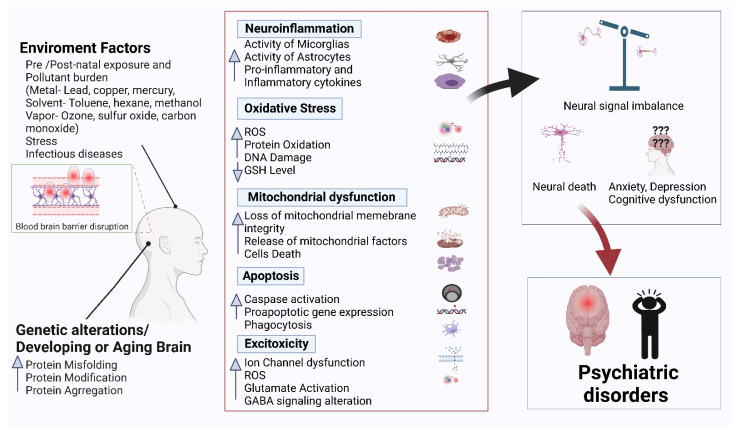
Common pathophysiology of psychiatric disorders.

**Figure 2:**
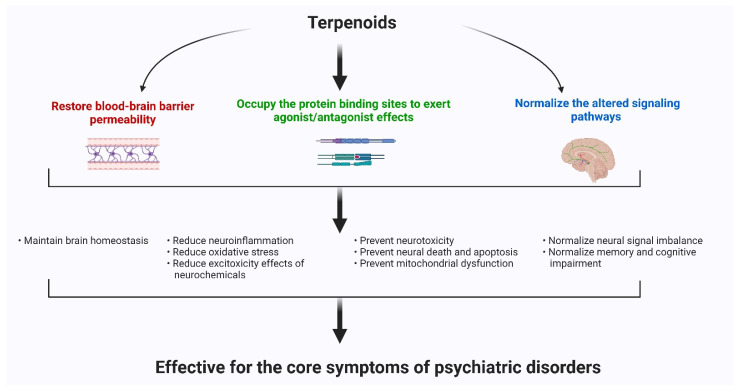
The major mechanisms of action of terpenoids.

Psychiatric disorders are patterns of behavioral alterations that cause significant distress or impairment of personal functions. The characteristic alterations in behavior and emotional state associated with malfunction and structural damage of the central nervous system have differentiated this class of pathology from common neurological disorders. In this review, we used the references of some of the major categories of disorders described in the Diagnostic and Statistical Manual of Mental Disorders (DSM) [9], the most widely used system for classifying psychiatric disorders and standardizing their diagnostic criteria. Psychiatric disorders are intertwined with multiple pathophysiological conditions, including oxidative stress, mitochondrial dysfunction, neuroinflammation, neuronal degeneration, and synaptic loss [10]. The selectivity and potency of terpenoids make them highly promising potential candidates for use in psychosis studies. In Figure 1, we describe the common pathophysiological changes associated with psychotic disorders and, in Figure 2, the overall major mechanisms of action of terpenoids.

The number of known terpenoids is currently exploding due to advances in isolation techniques, synthetic methods, and a plethora of biochemical diversity. Currently, symptomatic treatment does not alter the underlying course of neuropsychiatric disorders. Effective therapies for intricate psychiatric disorders are also limited by their extrapyramidal side effects. Terpenoids or terpenes have been used in traditional medicine for years as anti-inflammatory, antibacterial, anti-plasmodium, anticancer, and antioxidative medicine. These groups have also shown a positive contributions that have been proven in several preclinical studies on anxiety, depression and mood disorders. Therefore, new therapeutic approaches suitable for practical uses are urgently needed. Animal studies are invaluable to inform clinical research, to assess the necessity of further studies on a given topic and, most importantly, to assess the environmental exposure hazard in the evaluation of toxicological studies. Therefore, this review focuses on preclinical studies of natural or well-studied synthetic terpenoid derivatives and new terpenoid compounds with the potential to serve as novel, effective therapeutic interventions against psychiatric disorders.

## 2. Methodology

For the search strategy, we chose the following databases: PubMed, Science Direct, Cochrane Library, Embase, the Cumulative Index to Nursing and Allied Health Literature (CINAHL), Korea Med, ProQuest Full Text Library, Web of Science, and Wiley Online Library. We retrieved the data from the articles published until present. We used the search terms “terpenoids” or “terpenes” and “psychiatric disorders” (“psychiatric disorders” OR “psychiatric diseases” OR “neuropsychiatric disorders” OR “psychosis” OR “psychiatric symptoms”). All terms were searched independently in each database and in combination using the Boolean terms “AND” and “OR” as appropriate. Additionally, we retrieved some relevant articles through manual searches. Two researchers independently reviewed the retrieved articles for selection.

## 3. The Common Neuroprotective Mechanisms of Action of Terpenoids

### 3.1. Terpenoids Exert Neuroprotective Effects by Restoring Blood–Brain Barrier Permeability

The blood–brain barrier (BBB) is the primary metabolic interface between the peripheral blood supply and neural tissues or their fluid spaces. The key function of the BBB is to maintain central nervous system homeostasis and prevent undesirable harmful particles and chemicals from entering the brain [11]. The BBB protects the central nervous system from circulating pathogens, toxins, and xenobiotics, as well as restricts the migration of leukocytes and monocytes [12]. Several transporters and metabolites, such as P-glycoprotein and cytochrome P450 enzymes, located at the BBB, also protect the CNS [13]. These features greatly limit the transcellular and paracellular movement of molecules and cells across the cerebral microvasculature [11]. A highly specialized interconnected network of junctional complexes between adjacent endothelial cells along with high transendothelial electrical resistance (TEER) and relatively low pinocytotic activity confer the unique characteristics and functions of the BBB [14]. The loss of BBB vascular integrity increases the penetration of undesirable solutes, fluids, cells, pathogens, and toxins from peripheral circulation to the central nervous system. As a consequence, cerebral edema, cerebral hyperexcitability, and neuroinflammation occur and contribute to the clinical complications of many diseases [11]. BBB breakdown occurs in many neurological and neuropsychiatric disorders. The major routes for molecular transportation through the BBB include the transcellular and paracellular routes [13]. The structural disruption of the paracellular route is an important mechanism in the pathological state of BBB interruption. Therefore, disruptions in the assembly of junctional complexes and central endothelial cells decrease the integrity of the BBB and expose the CNS to systemic circulation. Pathological stimuli induce dysregulation of the structure of junctional complexes (both tight junctions and adherence junctions) and ultimately damage their specialized gating function [15,16]. Therefore, BBB integrity plays a pivotal role in the pathophysiological alteration of neuropsychiatric disorders.

Many inducers and mediators are involved in BBB breakdown, such as TNF-α, IL-1β, bradykinin, VEGF, MMPs, and oxidative stress [17,18,19,20]. Moreover, these disruptions can be mediated through the activation of PI3K, PKC, MAPK, and NF-κB, as well as the modulation of PPAR signaling transduction cascades [21,22,23]. The barrier properties of the BBB are of clinical significance from a neuropharmacological point of view for choosing the appropriate drug, as the barrier is largely seen as an obstacle for therapeutic compounds to reach their targets in the brain [24,25,26]. By targeting the abovementioned detrimental inducers and related signaling cascades, the stability of the BBB can be restored. Hence, the maintenance of BBB integrity is an excellent therapeutic target for neuropsychiatric drugs. Some novel therapeutics have been developed that show clinical efficacy and are able to restore the BBB. Numerous methods have been developed to aid the delivery of drugs to the CNS [26]. Most synthetic compounds at very high doses cause greater systemic off-target side effects. Therefore, nature-based products that elicit their activities in regulating transcription factors and signaling transduction cascades (e.g., PPAR, NF-κB, PI3K, PKC, and MAPK) are receiving interest in research to restore the architecture and function of the blood–brain barrier.

The protective effects of natural products against BBB breakdown are important mechanisms contributing to the clinical applications of some herbal medicines in the prevention and treatment of neuropsychiatric disorders. Their biological activities could be reflected by their chemical compositions, such as ginkgolide B from ginkgo, baicalein from skullcap, and tanshinone IIA from red sage, which are the most popular traditional medicinal products that exert neuroprotective effects [27]. Natural products possess several bioactive compounds that together have multicomponent, multitarget actions to address the intricacy of BBB breakdown. *Panax ginseng*, also known as ginseng, is a popular herbal medicine with neuroprotective potential. Its major terpenoid constituent, ginsenoide Rb1, has been reported as one of the major active constituents in ginseng. In rats, ginsenoside Rg1 has shown protective effects on BBB structural alteration and amended the severity of brain edema and BBB permeability [28]. *Ginkgo biloba* is a common complementary medicine that possesses anti-inflammatory, antioxidative, and vascular protective effects. Ginkgo is mainly known for its neuroprotective effects, and recent studies have verified its ability to protect the BBB [29]. Ginkgolide B, the major diterpenoid compound of the Ginkgo species, induced a protective effects on BBB permeability and brain edema in rats following hyperthermic brain injury.

### 3.2. Terpenoids Occupy the Receptor Protein Binding Sites to Exert their Agonist/Antagonist Effects

(a) MAO inhibition

The enzyme monoamine oxidase (MAO) metabolizes neurotransmitters (serotonin, dopamine, norepinephrine, tryptamine, etc.) and endogenous amines and xenobiotics. MAO is involved in catalyzing oxidative deamination of amines and neurotransmitters associated with oxidative stress and adverse pharmacological reactions (mood swing and depression) [30]. The two isoenzymes MAO-A and MAO-B have important roles both in the central nervous system and peripheral organs. MAO-A is associated with psychiatric conditions and depression, and MAO-B is involved in neurodegenerative diseases [31,32]. Oxidation of biogenic amines and neurotransmitters induced by MAO enzymes generates hydrogen peroxide (H_2_O_2_), oxygen radicals, and aldehydes. These phenomena increase the risk of cell oxidative injury. Therefore, inhibition of MAO may protect against oxidative stress and neurotoxins [33,34]. The inhibition of metabolizing enzymes may increase brain concentrations of their substrates, and thus, reduce disease symptoms of psychiatric disorders. Inhibitors of MAOs are considered to be an effective therapeutic intervention with neuroprotective and antidepressant effects. The expected therapeutic strategy in the treatment of depression and mood disorders is primarily pharmacological modulation of the monoamine system [34]. Because neurotransmitters (dopamine, norepinephrine, and serotonin) are metabolized by monoamine oxidase (MAO), inhibition of the enzyme may attenuate disease symptoms by balancing the concentration of neurotransmitters in the brain. Although synthetic MAO-A inhibitors are widely used as antidepressants, their prolonged use triggers adverse reactions (hypertension) [33]. Therefore, natural MAO inhibitors might be new alternatives for depression-related disorders. 

*Ixeris dentate* Nakai (compositae) is a perennial herb that has been used for different purposes as a folk medicine in Korea. Sesquiterpene lactone, glycosides, and flavone are the primary bioactive compounds isolated from this herb. In 2003, one preclinical study first showed that two flavone compounds, luteolin and cymaroside, exhibited MAO-B inhibition activity in adult Sprague–Dawley rats [35].

*Hypericum perforatum* L. (Guttiferae), commonly known as St. John’s Wort, is used as an alternative treatment for mild and moderate depression [36]. Phytochemical characterization has reported that hyperforin (a prenylated phloroglucinol) and hypericin (a naphthodianthrone) were the primary chemicals responsible for effects on health, although other biologically active constituents, for example, flavonoids, tannins, and different terpenes, have also been reported to exert antidepressant effects [37].

(b) Effects on GABAergic systems

GABA is an important inhibitory neurotransmitter that plays a pivotal role in the central nervous system. GABA-induced activation of ionotropic GABA receptors that are widely expressed in the brain exert a major inhibitory function. In the GABAergic terminal, GABA is formed from glutamate through an enzymatic reaction mediated by glutamic acid decarboxylase (GAD) and cofactor pyridoxal phosphate [38]. GABAergic dysfunction is crucial for the pathophysiological changes that occur in neuropsychiatric disorders. Therefore, targeting the GABAergic system is one of the mechanisms of antidepressants and mood stabilizers. These drugs affect several neurotransmitter systems, such as serotonergic, monoaminergic, and GABAergic systems [39,40]. Therefore, several lines of evidence support that low GABAergic function plays a key role in the pathophysiology of psychotic disorders, which are related to the dysfunction of multiple neurotransmitter systems [39]. Current antipsychotic treatments increase short-term levels of neurotransmitters in the brain, primarily affecting selective serotonin reuptake inhibitors (SSRIs), serotonin (5-HT) and noradrenaline (NE) reuptake inhibitors (SNRIs), and monoamine oxidase inhibitors (MAOIs) [41]. The consequence of long-term use of these drugs is the desensitization of some receptors, such as 5-HT_1A_ autoreceptors. The prevention of this inhibitory mechanism of 5-HT_1A_ receptor antagonists augments the neurochemical and behavioral effects of SSRIs [42,43,44]. Therefore, conventional antidepressants and anxiolytics, such as benzodiazepine (a GABA receptor agonist) and SSRIs, induce significant side effects, such as nausea and insomnia, fatigue, sedation, sexual dysfunction, headaches, and weight gain [41]. Hence, the increasing demand for alternative medicines that affect the GABAergic system is becoming important for alleviating the symptoms of these psychiatric disorders. Several alkaloids, flavonoids, terpenoids, and essential oils extracted from herbs, flowers, and other plant parts exert dynamic effects on GABAergic systems and have been widely used in traditional medicine as antidepressants and anxiolytics for a long time. The hydroxyl group of terpenoids has been primarily reported to exert effects on the GABAergic system [43,44].

Different essential oils are rich in terpenoids and phenylpropanoid derivatives. Essential oils (EOs) are extracted from herbs, flowers, and other plant parts and are comprised of volatile aromatic compounds. These volatile oils are concentrated hydrophobic liquids of natural products. The chemical components of essential oils could exert their biological actions via regulating the GABAergic system and inhibition of Na^+^ channels [44]. Dysfunction or deficiency of the GABAergic system has been implicated in neuropsychiatric disorders. The most common terpenoids found in essential oils are monoterpenes and sesquiterpenes [45]. The essential oils of *Anthemis nobilis* (chamomile), *Salvia sclarea* (clary), *Rosmarinus officinalis* (rosemary), *Lavandula angustifolia* (lavender), and *Rosa damascene* (rose) have been used as popular anxiolytic essential oils in Europe for years [44]. The antidepressant effects of EOs of chamomile, clary, rosemary, and lavender were assessed using a forced swim test (FST) in rats [46]. In this study, the authors reported that among the tested essential oils, clary oil exhibited the strongest antistressor properties in a FST. The fruits, leaves, and roots of *Piper guineense*, a popular medicinal herb, have diverse therapeutic uses for treating convulsion, rheumatism, and respiratory disease in African traditional medicine [47]. Inhalation of essential oils of *P. guineense* exert significant sedative and anxiolytic effects. The primary compounds of *P. guineense* EO are linalool and 3,5-dimethoxytoluene. Linalool might be the major compound that exhibits sedative effects partially via the GABAergic receptor system [47]. *Asarum heterotropoides* var. *mandshuricum*, from the Aristolochiaceae family, is a traditional Chinese medicine called Xixin or wild ginger which is used for pain and inflammation. The essential oils extracted from A. *heterotropoides* effectively attenuated depression-like behavior and increased brain expression of serotonin (5-HT) under forced swimming or immobilization stress [48]. *Melissa officinalis* L (lemon balm) is a traditional herbal medicine native to the Eastern Mediterranean region, Western Asia, and tropical countries, such as Brazil. This herb is widely used as a mild sedative, spasmolytic and antibacterial agent. The essential oils of *Melissa officinalis* are commonly used for sedative effect, improvement of cognitive functions, and other physiological actions [49]. Abuhamdah et al. showed that EO of M. *officinalis* reversibly inhibited GABA-induced currents in a concentration-dependent manner. Lavender (*Lavandula angustifolia*) is cultivated worldwide for its essential oils. These essential oils are used in perfumes, in modern aroma therapy, cosmetics, or in food processing [50]. Lavender inhalation has been used in folk medicine for the treatment of anxiety [44]. Another study reported that the EO of this lavender plant likely exerts its anxiolytic effect through serotonergic but not GABAergic neurotransmitters [44,51]. Linalool and linalyl acetate are the main bioactive components of the Lavender species that exerts therapeutics effects [52]. Thymoquinone is a major constituent of the essential oil of *Nigella sativa* seeds that have exhibited anti-anxiety activity in mice. In 2011, Gilhotra and Dhingra [53] demonstrated the anti-anxiety effect of thymoquinone by GABAergic and nitriergic modulation. Thymoquinone at 20 mg/kg exhibited anxiolytic effects by decreasing plasma nitrite level and reversed the decreased brain GABA materials in stressed mice.

(c) Dopamine D1 and D2 receptors

The dopaminergic system is involved in delayed maturation of the brain and plays an important role in stabilizing and integrating functions on neural circuits. Excessive neurotransmission of dopamine is associated with the pathophysiological alterations of many psychotic disorders and is a clinical hallmark of schizophrenia [54]. Dopamine receptors (DRs), which are G-protein coupled receptors, are becoming important as primary targets for developing drugs to treat neuropsychological disorders. DRs usually transfer signals into cells through guanine nucleotide-binding regulatory G-proteins [55]. DRs can be classified into two major subfamilies, D1 and D2 receptors. Antipsychotic drugs and dopamine both act on the same binding sites, but antipsychotic drugs do not bind or activate the G-protein [56]. It blocks the binding site of dopamine and prevents sodium ions from entering postsynaptic cells [57]. Antipsychotics known as neuroleptics are a class of compounds with a high affinity for several subtypes of dopamine receptors [58]. The chemical composition and structural variation of the antipsychotics allows them to bind to different subtypes of dopamine receptors without triggering the postsynaptic response that is exerted by dopamine under psychotic conditions. Neuroleptics can block dopamine receptors without triggering the ion channels to be opened or set off an action potential. Therefore neuroleptics are being administered to schizophrenic patients to aid in reducing excess levels of dopamine and this mechanism of action is very effective to alleviate the positive symptoms of this disorder [59]. The most commonly used “typical antipsychotic drugs” including phenotiazines, thioanthenes, butyrophenones, diphenylbutyl piperidines, and dihydroindolones, have a high binding affinity for the dopamine receptors and exert their therapeutic action. D2 dopamine receptors are present on both presynaptic cells and the post-synaptic membrane. Commonly used antipsychotic compounds can interfere with dopaminergic neurotransmission at different sites in both pre- and postsynaptic cells. Usually typical antipsychotics inhibit the dopaminergic neurotransmission in the limbic system and in the cerebral cortex [60]. These areas are important for controlling the motivational and emotional behaviors and thoughts. These mechanisms of action of antipsychotics are useful for alleviating the positive symptoms of schizophrenia and many psychotic conditions. 

Typical antipsychotics block all D2 receptors together with the other receptors involved in fine tuning of cognitive and motor association especially in the basal ganglia and cerebellum. Consequently, the inhibition of dopamine transmission causes exceedingly undesirable side effects such as tremors and akinesia [59]; additionally, it also inhibits the regular endocrine function, and elicits the anticholinergic, antiadrenergic, antihistaminic and antiserotonergic actions [57]. Another type of neuroleptic, known as “atypical antipsychotics,” exert very similar therapeutic effects as typical antipsychotics, but do not produce extensive level of side effects. The atypical antipsychotics demonstrate less affinity for D2 receptors than D3 and D4 dopamine receptors [60]. As the appearance of D3 and D4 receptors are limited to the neurons of the limbic system and cortex, therefore, new nature-based therapeutic interventions are needed to act on the vast region of the brain with safer effects. 

Limonene is a common terpene found in citrus fruits. This monoterpene is widely used as a flavor and fragrance. Limonene has been shown to exert anxiolytic effects, regulatory effects on neurotransmitters, and antinociceptive effects [61]. Previously it has been shown that limonene increased the metabolic conversion of dopamine and serotonin in the hippocampus and prefrontal cortex and striatum, respectively, suggesting that anxiolytic and antidepressant-like effects can include suppression of dopamine activity associated with increased serotonergic neurons through 5-HT_1_A [62]. To date, a very dynamic bioactive compound named (−)-stepholidine has been isolated from the Chinese herb Stephania, which is used as a drug and exhibits dual effects on D1 receptor agonists and D2 receptor antagonists. In addition, another preclinical study has shown that SPD has superior antipsychotic effects as compafed with conventional perphenazine [55].

### 3.3. Alteration of Several Signaling Pathways for the Protection and Survival of Neuronal Cells

Several signaling pathways are associated with neuropsychiatric disorders. Inflammation is a typical pathological feature involved in the progression of neuropsychiatric disorders. Microglia play an important role in the central nervous system and host defense mechanisms. Acute or chronic inflammatory processes cause long-lasting and excessive tissue damage in the central nervous system. Damaging stimuli such as wounds, infection, pathogens, or other foreign substances associated with brain injuries produce proinflammatory and inflammatory cytokines, such as IL-1β, IL-16, TNFα, and TGF-β_1_, which can induce microglia-mediated inflammation involved in acute or chronic neuropsychiatric disorders [63]. Therefore, anti-inflammatory pathways or reduced production of proinflammatory cytokines are therapeutic targets for antipsychotic drugs. The synthetic compounds of antipsychotic drugs provoke side effects with long-term use. Several studies have reported that the vast groups of terpenoids exert anti-inflammatory and antioxidative effects by altering inflammatory and oxidative stress-related pathways. Several groups of terpenes (D-limonene, α-phellandrene, terpinolene, boreol, linalool, and triterpene glycosides) have been reported to reduce the expression of TNF-α, IL-1, and IL-6 in in vivo models, such as Swiss mice, Wistar rats, and albino mice (BALB/C) [64].

The potent compounds bacoside A, bacopaside I and II, and bacosaponin of *Bacopa monnieri* have been reported to inhibit neuronal death by preventing AChE activity in vitro and in vivo, exerted anti-inflammatory and antidepressant effects and improved memory dysfunction in animal models [65]. In addition, α-pinene, d-limonene, camphene, myrcene, p-cymene, terpinolene, camphor, linalool, humulene, and β-caryophyllene have been reported as the group of terpenes that modulate the inflammatory process and oxidative stress [66]. In a former study, the antioxidant, anti-inflammatory, and neuroprotective activities of Ginkgolide B are reported and ascribed to downregulation of the Toll-like-receptor 4/NF-κB pathway [67].

## 4. Effects of Terpenoids on the Preclinical Studies of Psychiatric Disorders

### 4.1. Autism Spectrum Disorder (ASD)

Autism spectrum disorder (ASD), or autism, is a neuropsychiatric disorder with common features of language and communication deficits as well as stereotype/repetitive behavior [68,69]. A complex interplay of genetic and environmental insults is involved in the clinical manifestation of ASD. The prevalence of ASD has increased in the last few decades, and the disordered domains are attracting great concern due to their high prevalence along with social cost and large impact on the family [70]. Since the onset of disease occurs at a very early age, any dysregulation or dysfunction during the neurodevelopmental period is very important [71]. Therapeutic interventions that lower oxidative stress, normalize biomarkers, and restore the histoarchitecture of the forebrain are imperative for altered behavior. The oxidative biomarkers serotonin, glutathione, catalase, and nitric oxide and the histoarchitecture of the cerebellum determine and document the decreasing numbers of Purkinje cells, neuronal degeneration, and chromatolysis [72]. In this review, we identified that terpenoids and terpene-like compounds (diterpene, bacosides, resveratrol, and curcuminoids) play a vital role in animal models of ASD.

At early developmental stages, both prenatal and postnatal exposures to environmental insults, such as valproic acid (VPA), induce behavioral alterations similar to autistic symptoms [73]. *Bacopa monnieri* is a well-known plant that is extensively used in Ayurvedic medicine. Bacosides are important bioactive compounds isolated from *Bacopa monnieri*. Sandhya et al. [72] evaluated the effect of *B. monnieria* (L) on a VPA-induced autism model. On Day 12.5 of gestation, pregnant female rats were injected with VPA or saline (600 mg/kg i.p.). On postnatal day (PND) 21, VPA-induced male pups were separated and treated with *B. monnieria* (300 mg/kg/p.o.) from PND 21–35. Behavioral tests (nociception, locomotor activity, exploratory activity, anxiety, and social behavior) were performed during both adolescence (PND 30–40) and adulthood (PND 90–110). At the end of behavioral testing, animals were sacrificed for biochemical analysis (glutathione, serotonin, and nitric oxide) and histopathological examination. VPA remarkably altered normal behaviors, increased oxidative stress and serotonin levels, and altered the histoarchitecture of the cerebellum (decreased number of Purkinje cells, and chromatolysis) as compared with the control group. Treatment with B. *monnieri* significantly reversed behavioral alterations, normalized oxidative stress markers, and restored the histoarchitecture of the cerebellum [72]. Interestingly, postnatal VPA exposure induced oxidative stress and autism-like behavior in BALB/c mice [74]. Piperine, a major bioactive terpenoid isolated from *Piper nigrum* and *Piper longum*, has been demonstrated to possess neuroprotective [75], antioxidant [76], anxiolytic [77] and cognition-enhancing effects [78,79]. Pragnya et al., 2014, first revealed that treatment with piperine in a VPA-induced model significantly improved behavioral alterations, lowered oxidative stress markers, and restored cerebellar histoarchitecture in mice [74]. In conclusion, the findings of both studies suggest that phytochemicals of the terpenoid group ameliorate autistic symptoms, possibly due to their anti-anxiety, antioxidant, and neuroprotective properties.

The terpene-enriched essential oils of the *Salvia* spp. have anxiolytic and antidepressant properties [80]. This inexhaustible potential of the essential oils of *Salvia* spp. was used in a VPA-induced animal model [81]. The aerial parts were collected and dried away from sunlight. Essential oils of the dried powder of the *Salvia* spp. were extracted by hydrodistillation with water vapor. Thirty female (238.40 ± 18.70 g) and male (310 ± 48.60 g) Wistar rats were used. The inhalation method was used 60 min per day for 21 successive days for the *Salvia* spp. volatile oil-treated groups. The social interaction test (SIT), elevated plus maze (EPM), and FST were used to assess the autistic, anxiety, and depression status of the rats. The rats with ASD-like symptoms spent less time in the cage containing the control rat or the new rat and more time in the empty cage in the SIT than control rats. The 3% essential oil of *Salvia* spp. exerted an anxiolytic effect by decreasing the anxiety state created by valproic acid, by increasing the number and time spent in the open arms (1.75 ± 0.25; *p* = 0.009, F = 11.55) in the EPM test. The essential oil of *Salvia* spp. had an antidepressant effect on VPA 500 rats with severe depression (*p* = 0.00078 and F = 16.233). This oil significantly reduced the immobility time in the FST. This study confirms the pharmacological effect of the essential oil of *Salvia* genus plants in their psychotropic potential exploited in traditional medicine.

In addition, two well-known dietary sources from the terpenoid analog family, resveratrol and curcumin, were reported to show efficacy in the autism model as dietary supplements. We consider these two analog families of terpenoids because resveratrol is considered a nonflavonoid polyphenol compound containing a terpenoid backbone [82]. Biochemical analysis indicated that curcumin or curcuminoids are rich in terpene derivatives and contain predominantly monocyclic sesquiterpenes and oxygenated derivatives [83]. Considering their chemical structure, we also considered curcuminoids and resveratrol as terpenoid or terpene analogs and examined their neuropharmacological effects on ASD. Curcumin and curcuminoids are bioactive compounds in the spice plant turmeric, a member of the ginger family, and are widely used in traditional medicine in various inflammatory pathological conditions [84]. These compounds exert protective actions against various neurodegenerative and neuropsychiatric disorders [84]. They can cross the blood–brain barrier and target many degenerative pathways, including oxidative/nitrosative stress, mitochondrial dysfunction, and protein aggregation [85,86]. In the previous study, Al-Askar et al., showed the benefits of curcumin supplementation using a rodent model of VPA-induced autism-like syndrome. Curcumin plays a noteworthy therapeutic role in attenuating brain damage induced by prenatal VPA exposure in rats [87]. Concomitantly, resveratrol is receiving the attention of the scientific community due to its associated protective and therapeutic roles in several neurological diseases [82]. Resveratrol is a naturally occurring polyphenolic compound present in grapes, pines, peanuts, and red wine and is well known as an antioxidant and anti-inflammatory compound [88,89,90]. Considering this context, Bambini-Junior et al., [91] investigated the preventive effects of RSV on the autism-like social features of an animal model induced by prenatal exposure to VPA [91]. In the three-chambered apparatus test, supplemental RSV treatment in the VPA group counteracted the effects of VPA, restoring place preference conditioned by a conspecific and the tendency to explore a cage containing a rat in preference to an empty cage, suggesting that resveratrol could supplement a first line treatment to reduce the risk of VPA-induced autism-like behaviors.

### 4.2. Schizophrenia

Schizophrenia is a severe psychotic disorder characterized by continuous or relapsing episodes of psychosis. The diagnosis of schizophrenia is made based on a diverse set of characteristic signs and symptoms [92,93]. These signs and symptoms may vary, usually involving delusions, hallucinations, problems with thinking (cognition), or disorganized speech, and reflect an impaired ability to function and express emotion [94]. Current treatments for schizophrenia affect positive symptoms but have limited efficacy on prodromal symptom domains [95]. Antipsychotics that block the dopamine D2 receptor are approved for treatment of the psychotic symptoms of schizophrenia. However, these lines of treatments have the limitation of exerting adverse effects in treating the negative and cognitive symptoms of schizophrenia [95,96]. Therefore, there is a high demand for a new therapeutic intervention with fewer adverse effects and proven efficacy against the negative symptoms and obstinate cognitive dysfunction of schizophrenia. In that regard, Park et al., revealed the efficacy of oleanolic acid plant-derived pentacyclic terpenoids on schizophrenia-like behaviors in mice elicited by MK-801 [93]. A single administration of oleanolic acid blocked MK-801-induced hyperlocomotion in the open field test. The MK-801-induced prepulse inhibition deficit was also ameliorated by oleanolic acid. In the novel object recognition test, attention and recognition memory impairments were ameliorated by a single administration of oleanolic acid in the MK-801-induced group. Additionally, oleanolic acid normalized MK-801-induced alterations of signaling molecules, including phosphorylation levels of Akt and GSK-3β, in the frontal cortex. These results suggest that oleanolic acid may represent a potential candidate for the treatment of schizophrenia-like symptoms.

Ginseng, the root of *Panax ginseng* C.A. Meyer (PG) is a widely distributed and important medicinal plant in Asia. Ginsenosides, the most promising terpenoid phytochemicals, have beneficial effects on cognitive performance, memory, and neurodegenerative diseases. H.J.Kim et al., [97] showed the effect of PG extract on the offspring of maternal immune activation (MIA)-induced mice during prenatal development. The synthetic double-stranded RNA polyriboinosinic-polyribocytidylic acid [poly (I:C)]-induced animal model was used for model of behavioral deficits similar to those that occur in schizophrenia and other psychotic disorders. Pregnant mice (E9) were injected via the intravenous route with poly (I:C) (5 mg/kg) or vehicle control to induce MIA, and MIA offspring were subjected to oral vehicle or PG (300 mg/kg) treatment. In the acoustic startle response test, the MIA-induced sensorimotor gating deficit that was ameliorated by PG [97]. The results of the SIT (non-aggressive and/or aggressive pattern), open-field test (number/duration of behavior) and FST (immobility behavior) were significantly altered by PG extract in the MIA offspring. Western blot and immunohistochemical analyses showed that expression levels of certain neurodevelopmental proteins of the medial prefrontal cortex, including dihydropyrimidinase-related protein 2, LIM and SH3 domain 1, neurofilament medium, and discs large homolog-4, were declined in the untreated young but were improved in the PG-treated MIA young. In a prior study, [98] the antipsychotic effect of PG suggested that it might be useful in schizophrenia. As in the prenatal stress-induced schizophrenia model, PG extract significantly improved prenatal stress (PNS)-induced psychiatric effects. In the critical period of fetal brain development PNS is an important environmental risk factors for the development of schizophrenia in adult offspring. PG extract (300 mg/kg) was orally administered to the PNS-induced group. In the behavioral tests, grooming behavior in a social interaction test, line-crossing behavior in an open-field test, and swimming activity in a forced-swim test (FST) were administered to the PG-treated and non-treated PNS-induced stressed groups; in the PG-treated group the outcome of the tests was normalized. Western blot and immunohistochemical analyses showed that PG positively altered the downregulation of several genes following exposure to prenatal stress. These findings also provide supportive evidence that oral treatment with PG reduces the incidence of psychiatric disorders, such as schizophrenia.

Essential oil, α-pinene, an organic compound of coniferous trees, suppresses neuronal activity. Essential oil (α-pinene) is used as a safe food additive in the food industry. This essential oil contains organic terpene. Ueno et al., 2019 reported that inhalation of α-pinene in MK-801-induced schizophrenic mice exerted anxiolytic and antioxidant effects [99]. In this study, C57BL/6 male mice were used and were kept in a sealed container for 30 min to inhale α-pinene; then, MK-801 was injected. Behavioral tests (locomotor activity test, elevated plus maze test, Y-maze test, hot plate test, and tail suspension test) were performed 30 min after MK-801 injection. Due to the low molecular weight and lipophilic nature of α-pinene, it is absorbed from the nasal mucosa and crosses the blood–brain barrier. It stimulated the GABA receptor and acted on nerves and the brain. It induced calming and sedation-like activity in MK-801-induced schizophrenic mice.

### 4.3. Attention-Deficit/Hyperactivity Disorder

Attention deficit hyperactivity (ADHD) is a neuropsychiatric disorder of childhood and adolescence marked by an ongoing pattern of inattention and/or hyperactivity and impulsivity that interferes with function or development that is inappropriate for a person’s age [100]. Some individuals with ADHD also display difficulty in regulating emotions or problems with executive function. A number of combined treatment strategies have been suggested for ADHD. It is crucial to develop efficacious treatments for ADHD due to the risk of incurring comorbid conditions and abusing drugs [101,102].

Yuan et al. [103] demonstrated the effect of catalpol (iridoids are derivatives of monoterpenes), an active ingredient of *Rehmanniae radix preparata*. Catalpol is the most frequently used Chinese medicinal herb for the treatment of ADHD and and has been shown to affect behavior and neurodevelopment in spontaneously hypertensive rats (SHRs) [103]. In this study, SHRs were treated with vehicle, methylphenidate (MPH) (2 mg/kg/day, i.g.), and catalpol (50 mg/kg/day i.g). Wistar Kyoto (WKY) rats were used as the control group (vehicle). The findings revealed that catalpol in the MPH treatment group decreased the average speed, time spent in the central area, rearing times, and times visiting the central area; increased the immobility time of SHRs in the open field; and increased the number of visits to the target site (annulus) and the time spent in the target quadrant, in the Morris water maze test. Hematoxylin and eosin (H&E) staining showed that the disappearance of the nucleolus in the prefrontal cortex (PFC) and striatum of SHRs caused irregular neuronal arrangement and ruptured nuclear membranes. Moreover, immunofluorescence staining of NeuN and myelin basic protein (MBP) indicated that catalpol ameliorated neuronal loss and contributed to myelination. Finally, Western blot and immunostaining analyses suggested that several regulatory proteins were upregulated by catalpol treatment during prefrontal cortex development. Furthermore, brain-derived neurotrophic factor (BDNF), cyclin-dependent kinase 5 (Cdk5), p35, fibroblast growth factor (FGF) 21, and its receptor (FGFR) 1 were upregulated in response to catalpol. Although the pathway by which catalpol reduces neuronal loss remains unclear, catalpol effectively ameliorates hyperactive and impulsive behavior and improves spatial learning and memory in SHRs.

### 4.4. Bipolar Disorder

Bipolar disorder is a mental illness marked by extreme and unusual shifts in mood, energy, activity levels, concentration, and the ability to perform routine work confidently; cognitive impairments are considered the major manifest for bipolar disorders [104]. Bipolar disorder is typically diagnosed during late childhood or early adolescence. Symptoms vary from person to person, and these moods range from extreme “up” periods of elevated, irritable, or energized behavior (manic episodes) to extreme “down” periods of sadness or helplessness (depressive episodes); less severe manic periods (hypomanic episodes) can also occur [105,106]. Valproic acid (VPA) is an anticonvulsant used clinically for mood swing and bipolar disorders. Prolonged use of valproic acid effects major manifests of cognitive function. Umka Welbat et al. [107] investigated that cotreatment of asiatic acid and valproic acid reduced the risk of major cognitive impairment, similar to bipolar disorder. This study investigated if asiatic acid, a triterpenoid derived from the medicinal plant *Centella asiatica,* could prevent the spatial memory and neurogenesis impairments caused by VPA when used as a treatment for bipolar disorder. VPA treatment reduces histone deacetylase (HDAC) activity, resulting in neural stem cell proliferation and differentiation, which may explain the cognitive impairments produced in rodents and patients. Male Sprague–Dawley rats were injected with VPA (300 mg/kg) twice a day for 14 days (from days 15 to 28) and concomitantly with asiatic acid (30 mg/kg/day) for 28 days. Spatial memory was assessed using the novel object location (NOL) test, and hippocampal cell proliferation and survival were quantified by immunostaining for Ki-67 and bromodeoxyuridine (BrdU), respectively. The findings showed that VPA significantly reduced Ki-67- and BrdU-positive cell proliferation and survival in the subgranular zone (SGZ) of the hippocampal dentate gyrus (DG), and VPA-treated animals were unable to discriminate between objects in familiar and novel locations. However, these abnormalities were restored to normal levels by cotreatment with asiatic acid, which could be useful for preventing memory deficits in patients taking VPA in cases of bipolar or mood swing disorder.

### 4.5. Major Depression Disorder

Depression is a chronic disorder and serious medical illness that negatively affects a person’s daily life. It causes severe symptoms and affects how a patient feels, thinks, and acts [108,109]. This psychiatric disorder substantially contributes to mental impairment, physical disability, and socioeconomic burden, and has been predicted by the WHO to be the second leading cause of other disease conditions (panic disorder, social phobia, diabetes, etc.) [108,110,111,112]. The search for new drugs remains to be a desirable approach because currently applicable treatment regimens, including tricyclic antidepressants, selective serotonin reuptake, and monoamine oxidase (MAO) inhibitors, have limitations due to their side effects [109]. Several preclinical and clinical studies have found evidence that some phytochemicals are potent antidepressants [107,108,109,110,111,112,113,114,115].

*Achyranthes aspera* Linn., locally known as Chirchira, is an indigenous herb found in India that has been reported to exert antidepressant activity [113]. Similarly, *Asparagus adscendens* Roxb. of the family Liliaceae has been documented as an antidepressant and is most popularly used in Asian countries as an aphrodisiac and brain tonic [116].

Ginsenoside, a natural steroidal saponin in the terpenoid group, represents the primary active compound in the herb *Panax ginseng*, which is used in traditional medicine in China, Korea, Japan, and other Asian countries. Ginsenosides Rg1 [114,115,117], Rg2 [118], and Re [119] are being investigated as potential neuroprotective agents. Ginsenosides Rg1 and Rg2 are the most abundant and active ingredients of *Panax ginseng*, exerting pharmacological effects on the central nervous system, including protective effects against hippocampal neurotoxicity, and in a stress-induced rat model of vascular dementia [120,121]. Ginsenosides Rg1 has been reported to increase expression of neurotrophic factor such as BDNF and to enhance ERK and CREB phosphorylation in the prefrontal cortex. These bioactive compounds activate the hippocampal BDNF signaling pathway. Moreover, protopanaxatriol (PT)-type ginsenoside Re (GRe) has been shown to improve depression- and anxiety-like symptoms and cognitive impairment induced by restraint stress in rats [119]. Lee et al. reported that repeated immobilization stress caused reduced expression of BDNF in the hippocampus, which may be related to the pathogenesis of cognitive impairment [119]. Treatment with GRe significantly reduced depression and anxiety-like symptoms and cognitive impairment following repeated immobilization stress. GRe might exhibit antidepressant and anxiolytic activity, possibly through modulation of hypothalamic corticotrophin-releasing factor (CRF) and the noradrenergic system in the central nervous system. Therefore, GRe may be a useful material as an alternative medicine for treating stress-related disorders such as depression, anxiety, and cognitive impairment.

Additionally, oleanolic acid (OA) is the major active secondary metabolite isolated from *Pimenta pseudocaryophyllus.* The antidepressant-like effect of OA has been found to be attenuated by depletion of indolamine and catecholamines [110]. To investigate the antidepressant-like effects of OA derivatives, mice were treated orally with these compounds and subjected to the forced swimming test. Among all derivatives screened and using the FST, D1 displayed the most promising antidepressant-like effects, possibly acting on the 5-HT1A receptor without eliciting locomotor discoordination [110]. Moreover, a series of pentacyclic terpenoid molecules, such as boswellic acids, have been found to act as antidepressants, as described by [122]. A total of 30 Swiss albino male mice were used in the study, and commercial *Boswellia serrata* at three different doses, i.e., 50/kg, 100 mg/kg, and 200/kg, were orally administered. They were evaluated for antidepressant activity using the tail suspension test (TST) 60 min after drug administration. The findings of the study showed that *Boswellia serrata* had significant antidepressant activity at a dose of 100 mg/kg in acute models of depression.

Some new natural sources of terpenoid groups have een reported as new remedies for depression with reduced side effects, for instance, the squalene of microalgae *Aurantiochytrium* sp. in the Thraustochytriaceae family [123]. This oleaginous microorganism produces high levels of squalene, a biosynthesized triterpene hydrocarbon and a precursor for all steroids in animals and plants and has great potential as a renewable source of chemical products for drugs with antidepressant and other neuropharmacological properties [124,125]. Sasaki et al. provided the first evidence that *Aurantiochytrium* sp. reduced neuroinflammation through the modulation of neurotransmitter systems, and thus, possessed antistress and antidepressant effects [124]. In addition, dietary sources of volatile and fixed oils and flavoring components of the seeds *of Nigella* spp. confirmed the antidepressant-like activity [108].

*N. sativa* is rich in diverse phytoconstituents, monoterpenes, alkaloids, triterpenes, and saponins, with a wide spectrum of therapeutic activity [126]. The antidepressant effect was evaluated by [108] using the tail suspension test and the FST. Albino mice were orally administered *N. sativa* polar extract at two doses, 50 and 100 mg/kg. Phytochemical investigation of the two active fractions led to the isolation of quercetin-3-O-α-L-rhamnopyranoside 1, quercetin-7-O-β-D-glucopyranoside 2, tauroside E 3, and sapindoside B, which were isolated and found to be potential antidepressant constituents.

### 4.6. Anxiety Disorders

The term “anxiety disorders” refers to specific psychiatric disorders that involve extreme fear or worry. These disorders constitute a prodromal stage of other psychiatric disorders, and they represent one of the greatest challenges to mental health globally [127]. Currently, benzodiazepine receptor agonists are available to treat anxiety behavior, but more efficacious alternative or complementary medicine is still needed.

*Centella asiatica* is a psychoactive medicinal plant that has been used to treat anxiety for centuries in Ayurvedic medicine. Wanasuntronwong et al. [128] reported that the standardized extract of *C. asiatica* (ECa 233) containing triterpenoids demonstrated an anxiolytic effect. The test compound was orally administered, and the anxiolytic effect was assessed using an EPM, a light–dark box, and an open-field test. ECa 233 showed an anxiolytic effect in both acutely and chronically stressed animals, primarily accounting for madecassoside and asiaticoside. These findings suggested the possible utility of ECa 233 for the treatment of both acute and chronic anxiety in different neuropathological states. Other saponins and steroid compounds have been isolated from *Newbouldia laevis* (Bignoniaceae) and have been widely used in Nigeria for various therapeutic purposes [129]. This finding suggested that the hydroethanolic leaf extract of *N. laevis* elicits anxiolytic- and antidepressant-like activities mediated by dopaminergic enhancement. Moreover, phenolic compounds from *Acacia hydaspica* R. Parker, family Leguminosae possess several biological effects. Afsar et al., 2017, reported that A. *hydaspica* extract (AHM) and its derived fraction (AHE) produced antidepressant and anxiolytic effects by neutralizing reactive oxygen species (ROS) and improving brain antioxidant activity [130]. The observed activities might be attributed to the occurrence of different phytochemicals as a potential and promising class of therapeutics for the treatment of neurological disorders.

African oil palm (*Elaeis guineensis* Jacq.) is one of the most valuable medicinal plants in the traditional life of West Africa. Different parts of the plant are used in traditional medicine for therapeutic purposes (skin disease, bronchitis, menorrhagia, headache, migraine, and mental disorders) [131]. A recent preclinical study demonstrated by Islam et al. [132] reported that the bioactive compound squalene, an active compound of methanolic extract of African oil palm (MEEG), exhibited anxiolytic activity in the assessed behavioral tasks. Swiss albino male mice were orally administered MEEG at a dose of 200–400 mg/kg 30 min before the experiment, and the standard drugs diazepam and fluoxetine HCl were administered 15 min before the tests. MEEG treatment significantly normalized locomotor activity, anxiolytic activity in the open field test, and antidepressant activity in the forced swimming and tail suspension test. Squalene displayed anxiolytic activity by changing the function of neurotransmitters or the union of GABAergic pathways.

### 4.7. Others

Common volatile oils containing valerenic acid, isovalerenic acid, terpineol, and rosmarinic acid (diterpenes) are widely used in anxiety, insomnia, and depression. Sahu [133] reported that aqueous root extract of *Valeriana wallichii* (VW) improved sleep quality. This effect on sleep quality was dependent on the levels of monoamines in the cortex and brainstem and on the dose of the extract. At a dose of 200 mg/kg, oral treatment with VW significantly decreased levels of norepinephrine (NE) and serotonin (5-HT) in both the frontal cortex and the brainstem. It was concluded that VW root extract might be useful as an herbal therapeutic intervention for improving sleep quality and might be a potential intervention for the amelioration of sleep disorders. Rosmarinic acid (RA) is an important component of herbal treatments and has been validated to exert therapeutic effects in mood and depression disorders. Nie et al., 2014, conducted a study to assess the effects of RA on post-traumatic stress disorder (PTSD)-like symptoms. Administration of RA (10 mg/kg) alleviated PTSD-like symptoms in rats by inhibiting the ERK1/2 signaling cascade in the hippocampus [134]. These preliminary results provide evidence to support the use of RA treatment for PTSD in preclinical studies, but investigation and acceptable clinical trials might be needed to establish solid evidence of therapeutic efficacy. 

Different groups of terpenes, terpenoids, and cannabinoids of flower-producing plants (*Cannabis sativa*, *Lavender officinalis*, *and Citrus aurantium*) have some therapeutic ability. These biochemical compounds possess anxiolytic properties and have shown significant effects in several animal models assessed by the open field, elevated plus maze, and marble burying test [135]. Terpene coupled with cannabinoids has been reported to be used in mood and anxiety disorders. Mushrooms are considered to be an important therapeutic and nutritional food worldwide. Mushrooms are rich in different bioactive compounds such as terpenoids, sesquiterpenes, polyphenols, alkaloids, polysaccharide etc. Medicinal mushrooms are being used in traditional medicine for immune modulation, antioxidant, antimicrobial, and antitumor effects. Nowadays, these groups are receiving important attention in neurodegenerative and neurological studies. In a mixed psychiatric model of CNS depressant, anxiolytic and analgesic animal model, the aqueous and ethanol extract of *Ganoderma applanatum* showed potential CNS depressant, anxiolytic, and analgesic actions in the rodent studies [136].

Table 1 represents the bioactive terpenoids, their sources and mechanism of action in psychosis. Table 2 presents an overview of effects of terpenes or terpenoids in the preclinical studies of psychiatric disorders

## 5. Future Perspectives

In this wide-ranging review, we observed that, at present, the common groups of terpenoids or terpenes such as ginsenoside, bacosides, oleanolic acid, boswellic acid, asiatic acid, etc. have been extensively studied in preclinical psychiatric research. Among them, many compounds have been shown to meet the requirements for human use, while, for many others, the necessary experiments are underway. The main mechanisms of action of terpenoids on psychiatric diseases are: (i) to restore the blood–brain barrier integrity, (ii) to act as agonist or antagonists to the receptor binding sites of neurotransmitters, and (iii) to normalize the altered signaling pathways related to psychiatric disorders. The terpenoids groups exert their antipsychotic actions and amend the neuroinflammation process, and therefore, reduce oxidative stress level and apoptosis. They also restore mitochondrial dysfunctions and inhibit the release of excitotoxic materials that provoke the pathological condition of psychosis development.

Terpenoids are abundantly occurring compounds in natural products. Their easy obtainability has made them an effective tool in traditional medicine for years. Numerous terpenoid compound-based drugs (plant medicine) have been tested in several murine models of psychiatric disorders. Nonetheless, establishing translational models of psychiatric disorders is challenging. Many results have limitations with small case series or sample sizes, and some studies are often open label. To the best of our knowledge, information on toxicity studies of terpenoids in psychiatric disorders is limited, if not absent. It is important to consider the necessity of toxicological effects analysis for large clinical trials. It is also important to determine the safety and efficacy of natural terpenoid compounds as an alternative therapeutic approach in psychiatric disorders. In addition, a standardized procedure is needed to determine the purity and measure the concentration of these bioactive compounds to control the side effects in clinical uses. The appropriate dose selection will maximize the success in reversing the core and associated behavioral features of psychiatric disorders.

To the best of the author’s knowledge, psychiatric disorders have not been extensively studied as much as the common neurological or neuropsychiatric disorders, such as Alzheimer’s disease, Parkinson’s disease, epilepsy, and multiple sclerosis, possibly because psychiatric disorders are associated with behavioral and emotional states which are more complex to translate with precision in preclinical models. Preclinical studies are imperative for the future investigations of new drugs or therapeutic agents to evaluate and improve the outcomes. In this review, we emphasized preclinical studies to provide an overview on the current research status. These preclinical results might be a framework to quantify the experimental evidences to assess and improve the study design, rigor, or report the translational models of psychiatric disorders in future research and the potency of terpenoids as a therapeutic preference.

## 6. Conclusions

In this present review, we provide insight into the underlying mechanisms of action of terpenoids as antipsychotic drugs and we present evidence of the efficacy of terpenoids on associated psychiatric symptoms. These vast groups could be future effective regimens for treating the core symptoms of psychiatric disorders. We hope that this extensive review may be helpful to current research interests on terpenoids.

Regarding defining future research trends, it is important to consider the major limitations of the current research approaches. Correspondingly, from the chemical point of view, we suggest specific toxicological studies that include extensive preclinical trials, as these natural bioactive compounds are more complex in structure and functions than the typical synthetic compounds. Therefore, advanced studies on terpenoids would offer a substantial scope to design preclinical or clinical trials, for the discovery and development of terpenoids as novel and safer agents for therapeutic usage on psychiatric disorders.

## Figures and Tables

**Table 1:** Bioactive terpenoids, their sources and mechanisms of action in psychiatric disorders.

Psychiatric Disorders	Sources	Bioactive Terpenoids	Using Parts	Mechanisms of Action	Ref.
Autism Spectrum Disorder	*Salvia Spp*	Diterpenes	Essential oils	Facilitate the activity of GABA transaminase	[81]
	*Bacopa monniera (L)*	Bacosides A and Saponins A, B, C, and triterpenoid saponins	Whole plant extracts	Decrease hippocampal serotonin levels	[72]
	*Curcuma longa (Turmeric)*	Curcuminoids and co-occurring terpenoids	Root extracts	Decrease IL-6 level, suppresses the pro-inflammatory gene expression by blocking phosphorylation	[87]
	Grape, pine and Peanuts	Resveratrol (non-flavonoid polyphenol containing a terpenoid backbone)	Fruits	Regulate the level and activation of sirtuins (members of the class-III HDAC)	[91]
	*Piper nigrum Piper longum*	Piperine alkaloids and terpenes	Fruits	Reduced serotonin levels and oxidative parameters.	[74]
Schizophrenia	*P*. *vulgaris* var.lilacina	Oleanolic acid (plant-derived pentacyclic terpenoid)	Whole edible plants	Affected the metabolism of catecholamine and increased levels of 5-HT or NE due to MAO inhibition	[93]
	*Panax ginseng* C.A. Meyer	Ginsenosides (triterpene saponins)	Dry roots	Upregulate several neurodevelopmental genes	[98]
	*Panax ginseng* C.A. Meyer (PG)	Ginsenosides (triterpene saponins)	Dry roots	Increased certain neurodevelopmental proteins	[97]
	Coniferous tree *Eucalyptus* oil, camphor oil, *Bupleurum fruiticescens*, *Psidium,* and *Opunita humifusa*	α-Pinene (monoterpene)	Plants and essential oil	α-Pinene enhanced the function of the GABA-A receptor and played a vital role in GABAergic system	[99]
Attention-Deficit/Hyperactivity Disorder	*Radix preparata* (Rehmanniae)	Catalpol (iridoids are derivatives of monoterpenes)	Root extracts	Upregulate several regulatory proteins (BDNF), cyclin-dependent kinase 5 (Cdk5), p35	[103]
Bipolar Disorder	*Centella asiatica*	Asiatic acid (Triterpenoid)	Whole plant extracts	Asiatic acid could cross the blood–brain barrier and significantly restored oxidative stress marker	[107]
Major Depression Disorder	*Achyranthes Aspera* Linn.	Triterpenoid saponins	Leaves	Facilitated GABAergic system	[113]
	*Nigella sativa*	Monoterpenes, alkaloids, triterpenes, and saponins	Seeds	Inhibiting the reuptake of norepinephrine, serotonin, and dopamine	[108]
	*Boswellia serrata*	Boswellic acids are the series of pentacyclic terpenoid molecules	Dried resinous gum	*Boswellia serrata* showed high reducing ability of scavenging free radicals like NO, peroxide radical, O2., OH, DPPH (1,1-Diphenyl-2-picrylhydrazyl)	[122]
	*Pimenta pseudocaryophyllus*	Oleanolic acid	Leave essential oils	OA attenuated depletion of indolamine and catecholamine	[110]
	*Asparagus adscendens* Roxb. (Liliaceae)	Saponins, steroids, triterpenoids etc.	Roots	Reduced monoaminergic neurotransmitters, oxidative stress markers, and serum corticosterone levels.	[116]
	*Panax ginseng*	Ginsenoside Rg1(a natural steroidal saponins)	Roots and rhizomes and aerial parts	Increased the level of cAMP-response element-binding protein, brain-derived neurotrophic factor protein in the prefrontal cortex	[117]
	*Panax ginseng*	Ginsenoside Rg1	Roots and rhizomes and aerial parts	Exerted positive effects on neurogenesis within the hippocampus and increased BDNF protein levels	[115]
	*Panax ginseng* C.A. Meyer	Ginsenoside Rg1	Roots	Suppressed overexpression of proinflammatory cytokines and the activation microglia and astrocytes	[114]
	*Panax ginseng*	Ginsenoside Rg2	Roots and rhizomes and aerial parts	Activated the hippocampal BDNF signaling pathway	[118]
	*Panax ginseng* C. A. Mayer	GRe, Protopanaxatriol (PT)-type ginsenoside	Roots	Blocked the increased TH expression in the locus coeruleus (LC)	[119]
	*Aurantiochytrium* spp	Squalene (natural lipid belonging to the terpenoid)	Oleaginous microorganisms	Upregulated several genes involved in the neurotransmitter systems, dopaminergic and serotoninergic synapses	[124]
Anxiety Disorder	*Newbouldia laevis* (Bignoniaceae)	Saponins (Triterpenoid and steroidal glycosides, referred to collectively as saponins), steroids etc.	Leave extracts	Enhanced dopaminergic system	[129]
	*Centella asiatica*	ECa 233,triterpenoid glycosides including asiaticoside, madecassoside, asiatic acid and madecassic acid	Whole plant extracts	Facilitated GABAergic mechanism	[128]
	*Acacia hydaspica*	Polyphenols, terpenoids flavonoids, fatty acids etc.	Whole plant extracts	Reduced oxidative stress markers in the brain	[130]
	*Elaeis guineensis*	Squalene, stearic acid and different groups of terpenoids	Leaf	Enhanced function of neurotransmitter and normalized the GABAergic system	[132]
Sleeping disorder	*Valeriana wallichii*	Volatile oil (valerianic oil) containing valerenic acid, isovalerenicacid, and terpineol	Root extracts	Normalized monoamines neurotransmitters	[133]
Post-traumatic stress disorders (PTSD) or post traumatic mood disorder	Perillae Herbs	Rosmarinic acid (terpene (benzenediol abietane diterpene)	Plants and leaves	Facilitated neurogenesis and restored the ratio of neuron and astrocyte	[134]

**Table 2:** Effects of terpenes or terpenoids in the preclinical studies of psychiatric disorders.

Psychiatric Disorders	Terpene /Terpenoids	Induced Psychotic Condition by	Animals	Methodology	Pathophysiological Effects	Results	Ref.
Autism Spectrum Disorders	Diterpene	Valproic acid	Wistar Female & Male rats	3% essential oil (inhalation for 60 min per day) during 21 successive days	Altered GABAergic transaminase activity Increase monoamine oxidation	Essential oil of *Salvia*. spp shown antidepressant effect by inhibiting monoamine oxidase (MAOI) or selectively inhibiting serotonin and noradrenaline reuptake (SNRI)	[81]
	Bacosides A Saponin A,B,C triterpenoid saponins	Sodium valproate	Pregnant rats treated with sodium valproate and male pups	Pregnant female rat (E12.5) treated with sodium valproate Pups treated with aqueous extract of *B. monniera* (300 mg/kg, p.o)	Increased oxidative stress and serotonin level Decreased number of purkinje cells, neuronal degeneration	Aqueous extracts of B. *monniera* decreased oxidative stress markers and restored the VPA induced histoarchitecture alteration of cerebellum	[72]
	Curcuminoids and co-occurring terpenoids	Valproic acid (VPA)	Male Wistar Albino neonatal rats	Pregnant female rat (E12.5) treated with VPA pups treated with 1 mL of curcumin (1 g/kg b.wt) orally	Depletion of IFN-γ, serotonin, glutamineReduced glutathione, glutathione S-transferase, IL-6 in VPA exposed pups.	Improvement of brain toxicity level and brain dysfunction Improved in delayed maturation and abnormal weight of body and brain of VPA induced pups	[87]
	Resveratrol (non-flavonoid polyphenol containing a terpenoid backbone)	Sodium valproate	Female Wistar rats	Pregnant female rat (E12.5) treated with sodium valproate Resveratrol injected from E6.5 to E18.5 (3.6 mg/kg, s/c)	Prenatal VPA exposure effects on histone deacetylase (HDAC) inhibitory activity and several behavioral alterations such as social impairments and reduced sociability	RSV found to regulate the level and activation of sirtuins (members of the class-III HDAC)	[91]
	Piperine alkaloids and terpenes	Sodium valproate	BALB/c mice all neonatal pups	Piperine (20 mg/kg; orally) administered daily from PND 13 to 40. On PND 14, pups were injected VPA s/c	Postnatal VPA exposure at PND 14 increased oxidative stress, hyperserotonemia, and loss of Purkinje cell integrity in the cerebellum.	Piperine ameliorates sodium valproate induced behavioral deficits, increased serotonin and altered oxidative stress markers and Purkinje cell distribution in the cerebellum	[74]
Schizophrenia	Oleanolic acid (plant-derived pentacyclic terpenoid)	MK-801 (NMDA receptor antagonist)	Male ICR mice (6 weeks old; 25–30 g)	MK-801 (0.2 mg/kg, i.p.) Oleanolic acid (3,10 and 30 mg/kg, p.o)	Hyperlocomotion, memory impairment, PPI deficits Phosphorylation of Akt or GSK-3β in the frontal cortex	Oleanolic acid block MK-801-induced behavioral alterations and phosphorylation of Akt and GSK-3β in the frontal cortex	[93]
	Ginsenosides (Triterpene saponins)	Prenatal stress	Pregnant Sprague-Dawley rats	Powdered extracts of *Panax ginseng* (PG) during pregnancy	Abnormal axonal outgrowth, alter synaptic function and neurodevelopmental protein level in the offspring	Decreased the expression of neurofilament proteins and Dpysl2 following exposure to PNS	[98]
	Ginsenosides (Triterpene saponins)	Poly (I:C)	C57BL6/J mice (male & female)10 weeks males pups	Pregnant female mouse exposed to Poly (I:C) (5 mg/kg) on GD9 *Panax ginseng* (PG) (300 mg/kg/day, p.o) on postnatal day 35–65	Alterations in neurodevelopmental protein expression, including Nefm, Lasp1, Dlg4 and Dpysl2. Dpysl2.	PG treatment reversed the resulting MIA induced downregulation of neurodevelopmental proteins	[97]
	α-Pinene (monoterpene)	MK-801 (NMDA receptor antagonist)	C57BL6/J mice (male 15 Weeks)	Inhaled α- in a sealed container for 30 min	glutamatergic neurotransmission dysfunction and alter glutamatergic system	Inhaled α-Pinene alleviated MK-801 induced abnormal neural activity	[99]
Attention deficit hyperactivity	Catalpol (Iridoids are derivatives of monoterpenes)	Methylphenidate (MPH)	Young male Wistar Kyoto (WKY) Spontaneously hypertensive rats (SHR)	MPH (2 mg/kg/day, i.g.) Catalpol 50 mg/kg/day for 4 weeks	Structural and functional abnormalities of the brain in particular brain network and prefrontal striatal circuits mediating “executive” function	Catalpol exerted neuroprotective role by inhibiting neuronal apoptosis, increasing myelination and BDNF expression.	[103]
Bipolar Disorder	Asiatic acid (Triterpenoid)	Valproic acid	Male Spraque-Dawley rats	Asiatic acid (30 mg/kg/day) for 28 days VPA (300 mg/kg) twice a day from Day 15 to Day 28	VPA could alter cell proliferation and cell survival in the SGZ of the hippocampus	Asiatic acid enhanced hippocampal dendritic cell proliferation, synaptic connectivity and restored oxidative stress markers.	[107]
Major Depression Disorder	Triterpenoid saponins	Thermal induced pain	Wistar albino mice of either sex	5% Gum Acacia Suspension (400 mg/kg, i.p.) Diazepam (2mg/kg, i.p.)	Depression and anxiety in CNS is mediated by GABAA receptor complex. GABAergic activity in the brain reduce the inhibition activity, modified GABA synthesis and increase release of excitatory activity	Agonistic/facilitatory activities at GABAA receptor complex Increases the inhibitory chloride conductance and/or upregulate the effect of synaptically released GABA on the GABAA receptors	[113]
	Monoterpenes, alkaloids, triterpenes, and saponins	Physical stress by fasting	Swiss albino mice of both sexes	Aqueous extracts of *N*. *sativa* (50, 100 mg/kg, p.o.)	Dysfunction and dysregulation of neurotransmitters serotonin, norepinephrine, dopamine, glutamate and gamma-aminobutyric acid (GABA)	Antidepressant activity by inhibiting the uptake of norepinephrine, serotonin, and dopamine	[108]
	Boswellic acids (a series of pentacyclic terpenoid molecules)	Physical stress	Swiss albino male	Extract of *Boswellia serrata* (50, 100 and 200 mg/kg, p.o.)	Decreased levels of monoamines like serotonin, nor-adrenaline and dopamine in the central nervous system.	Antioxidant activity with high reducing ability of scavenging free radicals (NO, peroxide radical, O2., OH, DPPH)	[122]
	Oleanolic acid derivative	Pretreated with catecholamine and serotonin depletors	Male Swiss mice	Drugs (5–20 mg/kg, p.o. or i.p)	The increased MAO A reduce synaptic concentration of 5-HT, shorten the interaction of receptors and alter serotonergic transmission.	Inhibition of MAO A Increase synaptic concentration of 5-HT and potentiation of serotonergic transmission.	[110]
	Saponins, steroids, triterpenoids etc	Physical stress	Swiss Albino mice of both sexes	*A. adscendens* extract (AAE) (25, 50, and 100 mg/kg, i.p.) Imipramine (15 mg/kg; i.p) for 14 days	Hypothalamic-pituitary-adrenal axis dysfunction followed by elevated corticosterone levels and reduced monoamines	AAE enhanced the 5-HT and NE levels.	[116]
	Ginsenoside Rg1 (a natural steroidal saponins)	Chronic unpredictable mild stress	Male Wistar rats	Ginsenoside Rg1 (40 mg/kg, i.p.) for 5 weeks	Reduced BDNF in prefrontal cortex (PFC), amygdala and hippocampus	Increased expression of BDNF in PFC Enhanced ERK and CREB phosphorylation	[117]
	Ginsenoside Rg1	Chronic unpredictable mild stress	Male Wistar rats	Ginsenoside Rg1 (40 mg/kg, i.p.) for 5 weeks	Lower degree of antioxidant enzyme activity in the hippocampal along with elevating oxidative stress levels	Inhibited the upregulation of oxidative stress via the NOX1/NOX4 pathway	[115]
	Ginsenoside Rg1	Chronic unpredictable mild stress	Male Wistar rats	Ginsenoside-Rg1 (40 mg/kg, i.p) for 5 weeks	Increased Bcl-2 expression Decreased cleaved Caspase-3 and Caspase-9 expression in the vmPFC region	Rg 1 alleviated the overexpression of proinflammatory cytokines and activated the microglia and astrocytes	[114]
	Ginsenoside Rg2	Chronic mild stress	Adult male C57BL/6J mice	A single injection of Rg2 (10 and 20 mg/kg, i.p.) fluoxetine (20 mg/kg, i.p.)	Lower expression of brain derived protein and affect the survival and growth of neurons	Rg2 enhances hippocampal BDNF expression and produces antidepressant effects by inhibiting GSK3β activity	[118]
	GRe, f protopanaxadiol (PD)-type ginsenoside	Repeated immobilization stress	Adult male Sprague-Dawley	GRe (10, 20, or 50 mg/kg, i.p.) for 10 days	Deficits in memory and learning Decreased the BDNF mRNA expression in the rat hippocampus	Restored the decreased expression level of BDNF mRNA in the hippocampus	[119]
	Squalene (natural lipid belonging to the terpenoid)	Physical stress	Male ICR mice	EEA (100 mg/kg, p.o.); Imipramine (20 mg/kg, p.o.) for 14 days	Modulation of dopaminergic and serotoninergic synapses Increased proinflammatory-related genes expression	Upregulate the several genes expression of dopaminergic and serotoninergic synapses Reduction of proinflammatory-related genes	[124]
Anxiety Disorder	Saponins (Triterpenoid and steroidal glycosides, steroids etc.	Physical stress	Mice of either sex (15–25 g)	*N*. *laevis* (50, 100, 200, 400 and 800 mg/kg i.p)	Reduction of brain-derived neurotrophic factor protein and hampered neuronal growth.	Anxiolytic and antidepressant activities through enhancing the dopaminergic activity	[129]
	ECa 233, triterpenoid glycosides including asiaticoside, madecassoside, asiatic acid and madecassic acid)	Chronic immobilization stress.	Adult male ICR mice	ECa 233 (10, 30, 100 and 300 mg/kg, p.o.), madecassoside (16 mg/kg, p.o.), asiaticoside (10 mg/kg, p.o.)	Increase the physiological stress markers and serum cortisone level.	Reduced the serum cortisone level and improved the condition of anxiety in elevated plus maze test	[128]
	Polyphenols, terpenoids flavonoids, fatty acids etc	Chronic Unpredictable Mild Stress	Adult male Sprague Dawley rats	Methanol extract of *A*. *hydaspica* (200 mg/kg, p.o), fluoxetine (5 mg/kg, i.p) and diazepam (DZM) (1 mg/kg, p.o) for 7 days	Excessive Production of free radicals causes oxidative stress Alter serotonergic transmission and overall brain activity	Antidepressant activity implicated by increasing the level of noradrenaline and serotonergic transmission	[130]
	Squalene, stearic acid and different groups of terpenoids	Physical stress	Male Swiss albino mice	MEEG (200–400 mg/kg, p.o.)	Imbalance of neuro transmitters (5-HT, NE or DA) Disruption of GABAergic pathways Increased level of reactive oxygen species (ROS) in plasma and brain	Anxiolytic effect by opening of chloride channels activated by GABA and amplified the reaction of GABA receptor.	[132]
Sleeping disorders	Volatile oil (valerianic oil) containing valerenic acid, isovalerenic acid, and terpineol	Physical stress	Adult male Sprague-Dawley rats	VW (100, 200 and 300 mg/kg, p.o.) Diazepam (5 mg/kg, p.o.)	Increased monoamines in the cortex and brain stem region	VW water extract has a sleep quality Improving the serotonergic system Decrease the levels of monoamines in cortex and brainstem.	[133]
Post-traumatic stress disorders (PTSD)	Rosmarinic acid (terpene (benzenediol abietane diterpene)	Enhanced single prolonged stress (ESPS)	Male Sprague Dawley (SD) rats	RA (10 mg/kg, i.p)	Suppressed hippocampal cell proliferation Loss of pyramidal neurons Reduced neurogenesis in the dentate gyrus	RA improved hippocampal cell proliferation and increased pERK1/2 expression	[134]
